# New Insights on Antennal Sensilla of *Anastrepha ludens* (Diptera: Tephritidae) Using Advanced Microscopy Techniques

**DOI:** 10.3390/insects14070652

**Published:** 2023-07-20

**Authors:** Larissa Guillén, Lorena López-Sánchez, Olinda Velázquez, Greta Rosas-Saito, Alma Altúzar-Molina, John G. Stoffolano, Mónica Ramírez-Vázquez, Martín Aluja

**Affiliations:** 1 Red de Manejo Biorracional de Plagas y Vectores, Instituto de Ecología, A.C.—INECOL, Clúster Científico y Tecnológico BioMimic, Carretera antigua a Coatepec 351, El Haya, Xalapa 91073, Ver., Mexico; alma.altuzar@inecol.mx; 2 Red de Estudios Moleculares Avanzados, Instituto de Ecología, A.C.—INECOL, Clúster Científico y Tecnológico BioMimic^®^, Carretera antigua a Coatepec 351, El Haya, Xalapa 91073, Ver., Mexico; lorena.lopez@inecol.mx (L.L.-S.); olinda.velazquez@inecol.mx (O.V.); greta.rosas@inecol.mx (G.R.-S.); m.ramirez@ciencias.unam.mx (M.R.-V.); 3 Stockbridge School of Agriculture, University of Massachusetts, Amherst, MA 01003, USA; stoff@umass.edu

**Keywords:** *Anastrepha ludens*, Tephritidae, olfaction, antennal ultrastructures, sensilla, sensory pit, hourglass-pore

## Abstract

**Simple Summary:**

In insects, including tephritid fruit flies, some of which are notorious pests of commercially grown fruit, the antenna harbors the sensilla responsible for the perception of odors (chemicals carried by air), temperature, humidity, and movement. As one of the methods used to monitor and control these agricultural pests is using traps baited with attractive odors, or toxic bait sprays, both of which an adult fly detects through the antenna, the study of this organ is crucial in understanding the behavior of the insect and applying this information in its environmentally friendly control/management. In this study, we detected up to 16 different subtypes of sensilla and various other hitherto unknown structures with the help of various types of microscopes in the antenna of the Mexican Fruit Fly, *Anastrepha ludens*, a pest of citrus and mango. We describe these sensilla/structures and suggest possible functions. As other researchers have previously worked on this topic, we made a special effort to uniformize the criteria used to classify these key structures, update the terminology, and better describe each sensilla with the help of detailed photographs.

**Abstract:**

Using light, transmission, scanning electron, and confocal microscopy, we carried out a morphological study of antennal sensilla and their ultrastructures of the Mexican Fruit Fly *Anastrepha ludens* (Loew), an economically important species that is a pest of mangos and citrus in Mexico and Central America. Our goal was to update the known information on the various sensilla in the antennae of *A. ludens*, involved in the perception of odors, temperature, humidity, and movement. Based on their external shape, size, cuticle-thickness, and presence of pores, we identified six types of sensilla with 16 subtypes (one chaetica in the pedicel, four clavate, two trichoid, four basiconic, one styloconic, and one campaniform-like in the flagellum, and three additional ones in the two chambers of the sensory pit (pit-basiconic I and II, and pit-styloconic)), some of them described for the first time in *A. ludens*. We also report, for the first time, two types of pores in the sensilla (hourglass and wedge shapes) that helped classify the sensilla. Additionally, we report a campaniform-like sensillum only observed by transmission electronic microscopy on the flagellum, styloconic and basiconic variants inside the sensory pit, and an “hourglass-shaped” pore in six sensilla types. We discuss and suggest the possible function of each sensillum according to their characteristics and unify previously used criteria in the only previous study on the topic.

## 1. Introduction

The antenna is considered the major sensory organ of insects [1] because it contains the main receptors involved in perceiving odors, movement, temperature, and humidity [2]. The receptors are located in sensilla, which have been classified according to their function (i.e., olfactory, mechanoreceptors, or thermohygroreceptors), external morphology (e.g., clavate, trichoid, basiconic, campaniform, and styloconic), cuticle texture (i.e., multiporous pitted sensilla (MPS), no-pore sensilla (NPS), multiporous grooved sensilla (MPGS)), size, thickness-walled cuticle (i.e., thick-walled and thin-walled) and a combination of some of these characteristics [3,4,5,6,7].

In the case of fruit flies (Diptera: Tephritidae), different authors have reported distinct types of sensilla in various economically important species. For example, for *Ceratitis capitata* (Wiedemann), Levinson et al. [8] reported three types of sensilla, whereas Mayo et al. [9], Dickens et al. [5], and Bigiani et al. [10] reported four. Four types were reported in *Anastrepha* (formerly *Toxotrypana*) *curvicauda* (Gerstaecker) [11], *Anastrepha ludens* (Loew), *Bactrocera cucurbitae* (Coquillett) and *Bactrocera dorsalis* (Hendel) [5]. Recently, Perre et al. [12] also reported four types of olfactory sensilla in *Anastrepha obliqua* (Macquart)*, Anastrepha bistrigata* Bezzi, *Anastrepha grandis* (Macquart)*, Anastrepha serpentina* Wiedemann, *Anastrepha* sp.2 aff. *fraterculus* (s. Selivon)*, Anastrepha sororcula* Zucchi, *Anastrepha montei* Lima, and *Anastrepha pickeli* Lima. Additionally, Hu et al. [13] reported six types of sensilla in *B. cucurbitae* and *B. dorsalis*, and other authors reported five in *Eurosta solidaginis* Fitch [14], six in *Bactrocera oleae* (Rossi) [15], *Bactrocera tryoni* Froggatt [13,16], *Bactrocera tau* (Walker), *Bactrocera minax* (Enderlein), *Bactrocera diaphora* (Hendel), *Bactrocera scutellata* (Hendel) [17], and *Anastrepha fraterculus* (Wiedemann) [7], seven in *Bactrocera zonata* (Saunders) [18,19], and ten types in *A. serpentina* Wiedemann [20]. The challenge one faces with this specialized literature is that the use of different study techniques/methodological approaches for these structures results in different classifications and terminologies for naming them, a fact that can generate confusion.

*Anastrepha ludens*, the Mexican fruit fly, is an economically important species that attacks citrus and mangos. Despite its significant status as a pest, the antenna have been little studied. The only known study is the one by Dickens et al. [5], who, using Scanning Electronic Microscopy (SEM) and Transmission Electronic Microscopy (TEM), reported, according to the cuticular texture and internal morphology, four types of sensilla (thick-walled MPS, thin-walled MPS, MPGS and NPS) in the antennal flagellum of males and females.

In preliminary observations on the antennae of wild *A. ludens* flies, we recognized some structures that were not mentioned in the work of Dickens et al. [5], which could be important in future electrophysiological studies searching for chemical compounds to develop attractants. We also recently studied the broad morphology and proteomics of the antennae of this pestiferous species, with the aim of better understanding the response to a potent commercial attractant [21]. Considering the above, we report on an in-depth morphological analysis of the sensilla present in the flagellum and sensory pit in the antenna of mature and immature *A. ludens* females and males using light, SEM, TEM, and confocal microscopy techniques. We also update the terminology in the context of the current nomenclature and suggest the types of sensilla that could be associated with the chemical reception of various volatiles.

## 2. Materials and Methods

### 2.1. Insects

For SEM and TEM studies, we used wild *A. ludens* flies originating from white sapote fruit (*Casimiroa edulis* La Llave and Lex.), one of the *A. ludens* native hosts collected in Xalapa, Veracruz, Mexico. For confocal microscopy images, we used Laboratory-reared flies maintained at the Red de Manejo Biorracional de Plagas y Vectores at the Instituto de Ecología A.C. in Xalapa, Veracruz [22]. This colony is periodically refreshed with wild material, so we felt justified in using some specimens, as morphological changes in the antenna have not been reported in lab-reared flies.

Newly emerged and 15–20-day old *A. ludens* females and males were used to identify the antennal sensilla using three microscopy techniques (confocal, SEM and TEM). In the case of sexually mature flies (15 days old), they were kept from their emergence as adults (from pupae) until their use in 30 × 30 × 30 cm Plexiglass cages with food ad libitum (mixture 3:1 of sugar and protein) and water in a laboratory at a temperature of 27 ± 1 °C and RH of 70 ± 5%. We kept low numbers of flies in these enclosures to avoid damage to the antennae or contamination through excessive dust or other materials.

Considering that there are different terminologies for identifying and classifying fly antennal sensilla in the literature, we reviewed previous publications and summarized them to homologize the terminology/nomenclature being used. We decided to use the classical nomenclature, where the classification scheme is based on the external shape of the sensilla in combination with the cuticular texture terminology used by Giannakakis and Fletcher [16].

### 2.2. Transmission Electron Microscopy (TEM)

**Figure 1:**
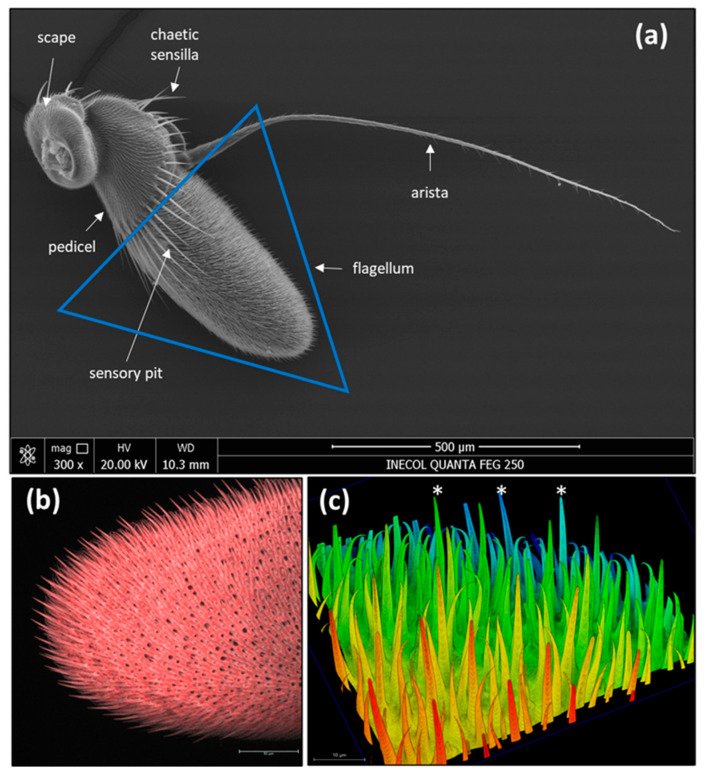
(**a**) Scanning electron micrograph of an overall view of the antennae of *A. ludens*; (**b**) Confocal image of the apical part of the flagellum showing the distribution of different sensilla types represented by black holes of different sizes; (**c**) Confocal image showing different types of sensilla (the longest ones marked with white asterisks are trichoid sensilla); further details in Video S1.

Antennae of five females and males of both ages were fixed over a week in a mixture of 2.5% glutaraldehyde and 2.0% paraformaldehyde in phosphate buffer at pH 7.4 [23]. Samples were then post-fixed in 1.0% OsO4 for 2 h and then dehydrated using a graded ethanol series (30–100%) for 10 min at each concentration. Heads with antennae were mounted in LR–white resin polymerized at 50 °C for 24 h inside jelly capsules (EMS^®^, Hatfield, UK). Ultrathin longitudinal sections around the antenna (blue peripheral line in Figure 1) of 70 nm were cut with a Leica EMUC7 ultramicrotome; then, basal, medial, and apical sections of the flagellum were analyzed. The slides were placed on a 200-copper mesh (EMS^®^) and stained with 2% uranyl acetate and lead citrate [24]. Samples were examined with a JEM-1400 PLUS transmission electron microscope (JEOL Ltd., Tokyo, Japan) and photographed using a GAT-830.10U3 camera (GATAN Inc., Pleasanton, CA, USA).

### 2.3. Scanning Electronic Microscopy (SEM)

Antennae of five females and males of both ages were fixed in a Karnovsky solution [23] for at least a week. Once fixed, specimens were rinsed three times in phosphate buffer at pH 7.2, and then dehydrated using a graded ethanol series (30, 50, 70, and 90%) for 30 min at each concentration and three times with absolute alcohol for 15 min. They were then dried in a critical point dryer (Quorum K850, Quorum technology, UK), followed by attachment to aluminum stubs using a carbon adhesive before coating with gold in a sputtering Quorum Q150 RS [25]. The preparations were studied and photographed with a FEI Quanta 250 FEG scanning electron microscope (FEI Co., Brno, Czech Republic).

### 2.4. Confocal Microscopy

Antennae of five females and males of both ages were fixed in 4% paraformaldehyde and PBS (0.2 m/7.2 pH). Subsequently, they were placed in a 10% potassium hydroxide solution to remove other tissue, mostly fat bodies. The antennae were stained with Congo Red (Sigma-Aldrich, Steinheim, Germany) dissolved in 70% ethanol and incubated at room temperature for 72 h. The samples were gradually dehydrated in ethanol (70% to 100%). The antennae were mounted with CytosealTM 60 mounting media (Richard-Allan Scientific™ Thermo Scientific™).

Imaging and rendering: Serial optical sections were obtained at 0.2 mm intervals on a TCS-SP8+STED (Leica Microsystems GmbH, Wetzlar, Germany) confocal microscope with an HCX PL APO 40x/1.30 OIL CS2 objective and HCX PL APO 63x/1.40 OIL CS2 objectives. A laser line of 488 nm was used for imaging the Congo-Red-stained cuticle, the laser power was set to 30% and the emitted fluorescent light was detected in the range from 613 nm to 683 nm.

## 3. Results

As previously reported for *A. ludens* and other tephritid flies [7,8,12,15], the antennae have three segments: scape, pedicel, and flagellum (also called funiculus or post pedicel), covered with different types and subtypes of sensilla and microtrichia (Figure 1, Video S1). The comparison of each antennal-segment size, measured by its length and width, indicates no differences between females and males, except for the width of the flagellum (Table 1). As Dickens et al. [5] originally reported, the antenna also has an arista inserted on the dorsal–proximal end of the flagellum and a sensory pit (also named olfactory pit) present on the dorsal–proximal surface of the flagellum (Figure 1).

**Figure 2:**
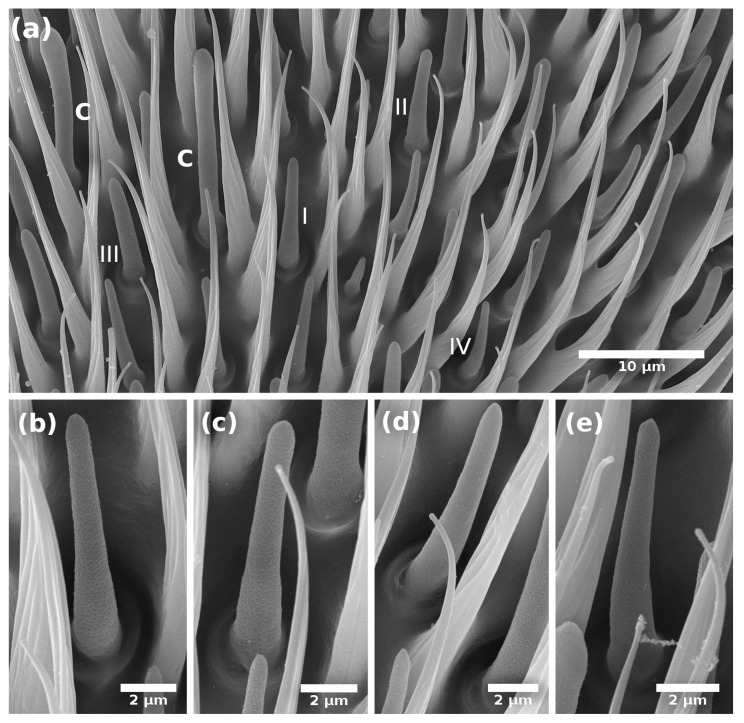
Scanning electron micrographs of the basiconic sensilla on the flagellum (apical segment) of *A. ludens* male: (**a**) Segment showing basiconic sensilla subtypes I–IV; (**b**) Subtype I; (**c**) Subtype II; (**d**) Subtype III; (**e**) Subtype IV.

**Figure 3:**
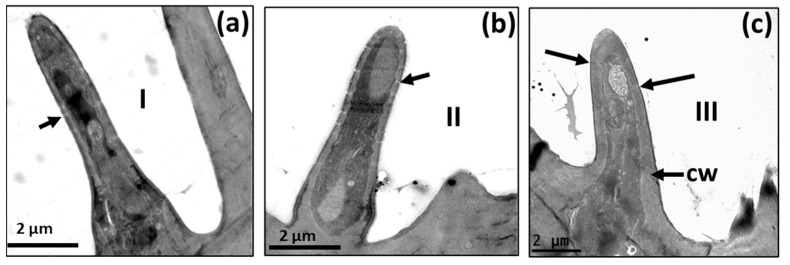
Transmission electron micrographs of a longitudinal section of the basiconic sensilla on the flagellum of *A. ludens* antenna showing cuticle wall [cw] and pores [arrows]: (**a**) Subtype I with thin-cuticle wall and pores of 15-day-old male; (**b**) Subtype II with thin cuticle wall of 15-day-old male; (**c**) Subtype III with thick cuticle wall of 15-day-old female.

**Figure 4:**
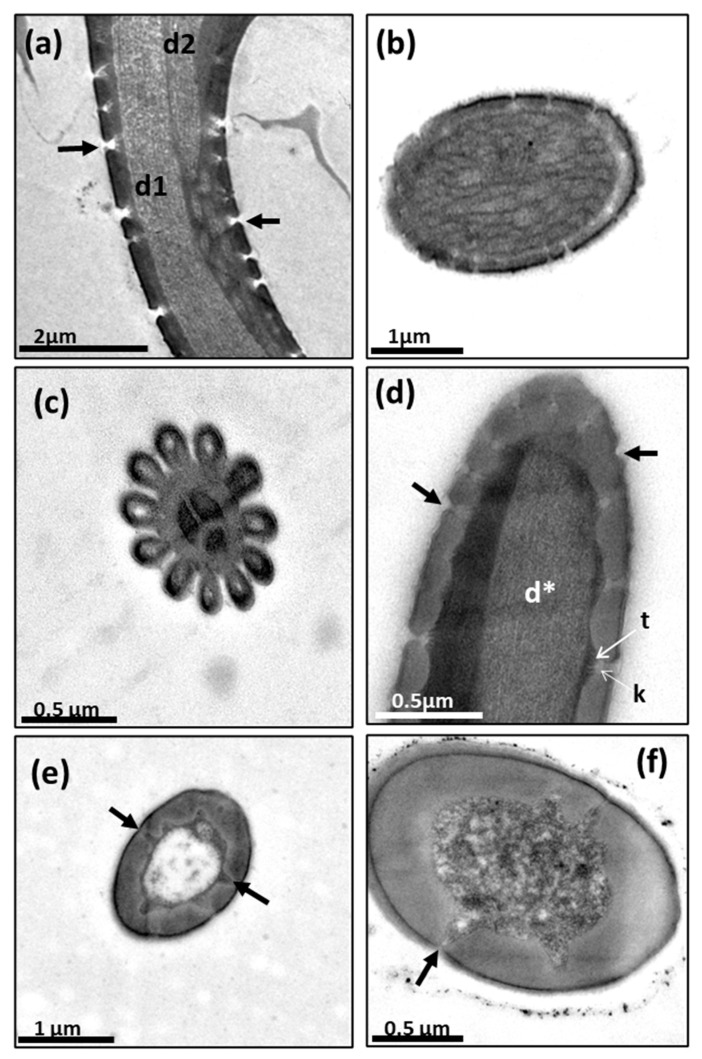
Transmission electron micrographs of sensilla in the antenna of *A*. *ludens* individuals: (**a**) Close-up of the longitudinal section of a trichoid sensillum showing in its internal middle part two dendrites [d1 and d2]. Note the hourglass pores (arrows), the kettle or pore pot, and the pore tubules through which the volatile molecules disperse towards the sensilla lymph; (**b**) Cross-section of the distal part of a clavate sensillum showing the lamellar dendrites inside and its cuticle interrupted by pores; (**c**) Cross-section of a styloconic sensillum showing double-wall, three well-defined dendrites in the central part and 11 digitiform projections similar to what Dickens et al. [5] reported; (**d**) Longitudinal section of a basiconic sensillum on its distal part, showing a dendrite (d*) inside and the pores in the cuticle with an hourglass shape (arrows), the kettle (k), and the tubules (t); (**e**,**f**) Transverse section of two thick-walled sensilla with wedge-shaped pores (arrows) like the one found in the basiconic thick-walled sensillum subtype III and the clavate subtype III.

**Figure 5:**
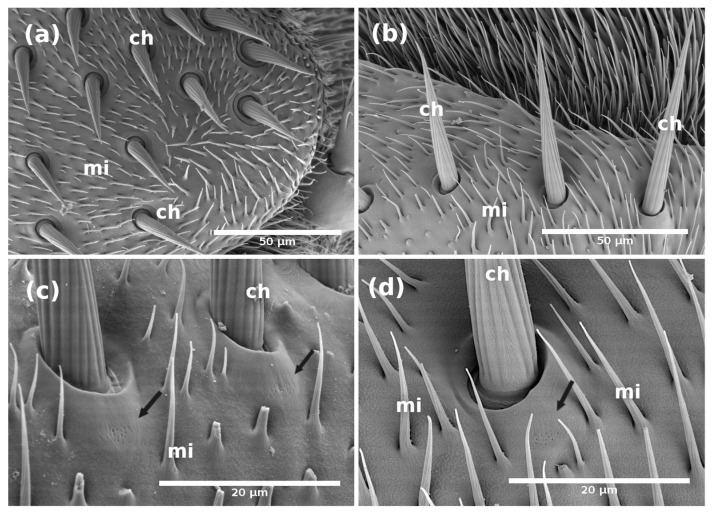
Scanning electron micrographs of chaetica sensilla [ch] and microtrichia [mi] on the pedicel of *A. ludens*: (**a**) Aerial view showing the distribution of chaetica sensilla and microtrichia; (**b**) Lateral view of the marginal part of the pedicel, where it is possible to see the cone shape of the chaetica sensilla; (**c**,**d**) Close-up of the base of chaetica sensilla showing the longitudinally ridged cuticle, the socket, and the groups of tiny pores in the socket base, denoted by black arrows.

**Figure 6:**
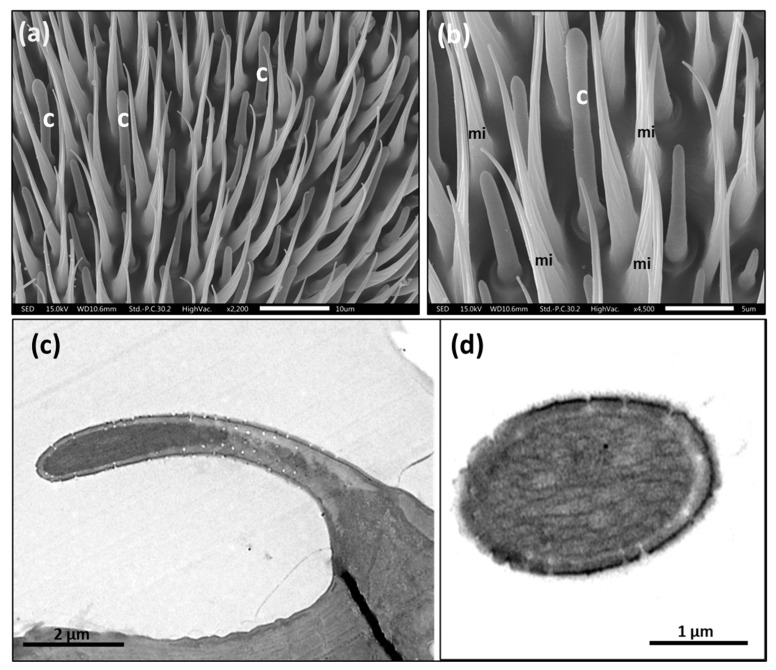
(**a**) Scanning electron micrograph showing the distribution of clavate sensilla (details under ‘c’) in a flagellum segment of a 15-day-old female *A. ludens*; (**b**) Scanning electron micrograph with a close-up of clavate type sensillum surrounded by microtrichia (mi) and other sensilla types; (**c**) Transmission electron micrograph of a clavate sensilla subtype II characterized by a thin cuticle with multiple pores; (**d**) Transmission electron micrograph showing a transversal section of a thin-walled clavate sensillum in newly emerged males (0 days).

**Figure 7:**
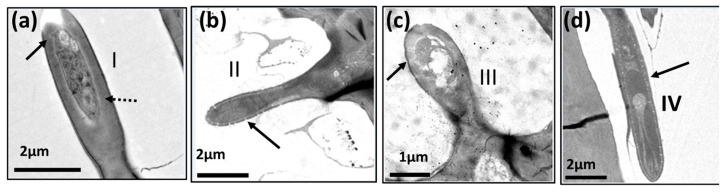
Transmission electron micrographs of longitudinal sections of clavate sensilla on the flagellum of *A. ludens* antenna: (**a**) Subtype I sensillum with thick cuticle wall (dotted arrow) and with wedge-shaped pores (black arrow) of 15-day-old male; (**b**) Subtype II sensillum with thin cuticle wall, hourglass-shape and multiple pores (arrow) of 15-day-old female; (**c**) Subtype III sensillum, the smallest of the subtypes, with thick cuticle wall and wedge-shaped pores (arrow) of 0 day-old female; (**d**) Subtype IV sensillum with thin cuticle wall (arrow) and multiple hourglass-shape pores (arrow) in a 15-day-old male (as shown in Figure 4a).

**Figure 8:**
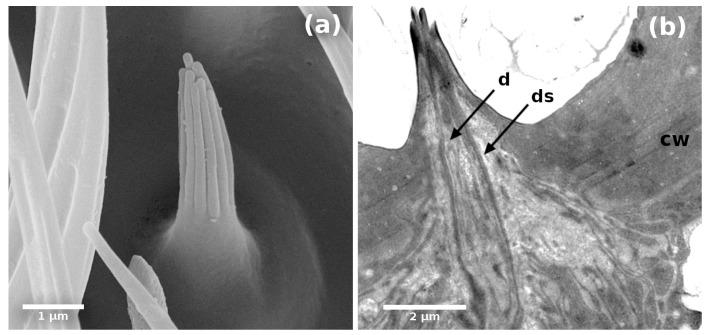
(**a**) Scanning electron micrographs of styloconic sensillum of *A. ludens*; (**b**) Transmission electron micrographs of styloconic sensillum showing an electron-dense dendritic sheath [ds], dendrites [d], and the cuticle wall [cw].

**Figure 9:**
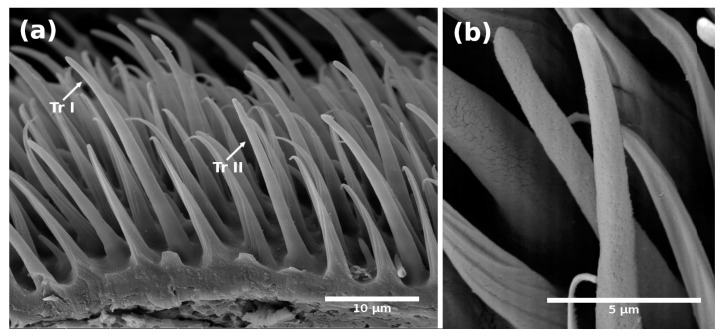
Scanning electron micrographs showing: (**a**) Trichoid sensilla among microtrichia; subtype I (Tr-I) sharply tipped and subtype II (Tr-II) blunt tipped on the flagellum of a 15-day-old *A. ludens* female; (**b**) Close-up of trichoid sensilla showing multiple pores on the surface.

**Figure 10:**
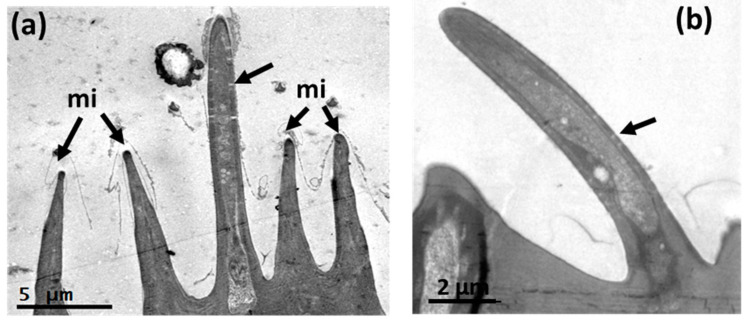
Transmission electron micrographs of a longitudinal section of the trichoid sensilla on the flagellum of an *A. ludens* adult antenna: (**a**) Subtype I with sharp-tip among microtrichia (mi) in a newly emerged female (black arrow points to hourglass-shaped pores); (**b**) Subtype II with a blunt tip (black arrow points to hourglass-shaped pores) in a 15-day-old female.

**Figure 11:**
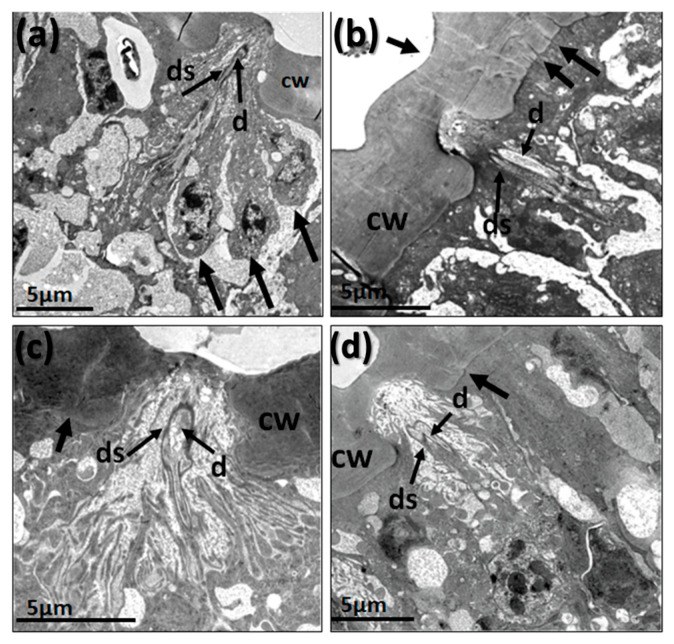
Transmission electron micrographs of the ultrastructure of a campaniform-like sensillum in *A. ludens*: (**a**) Longitudinal section of male sensilla showing three cells (black arrows) close to the sheath surrounding the sensilla’s dendrite and cuticle wall [cw]; (**b**–**d**) Longitudinal section of like-campaniform sensillum of females showing two sensory dendrites [d] with dendrite sheath [ds] and numerous channels (black arrows).

**Figure 12:**
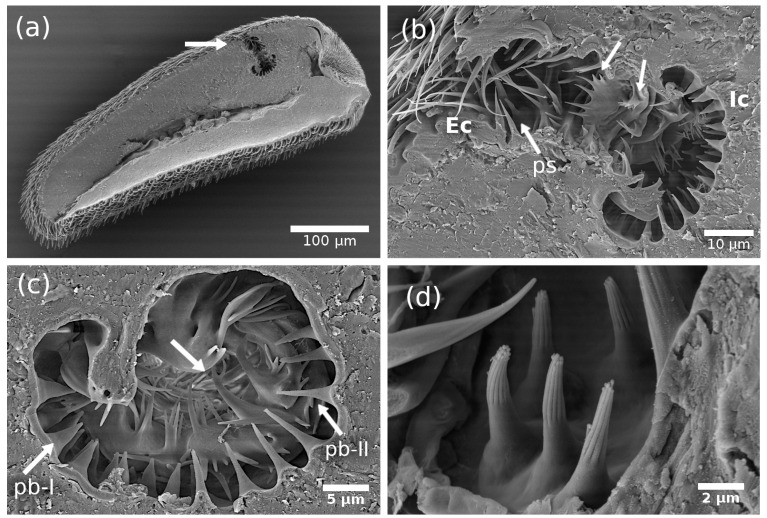

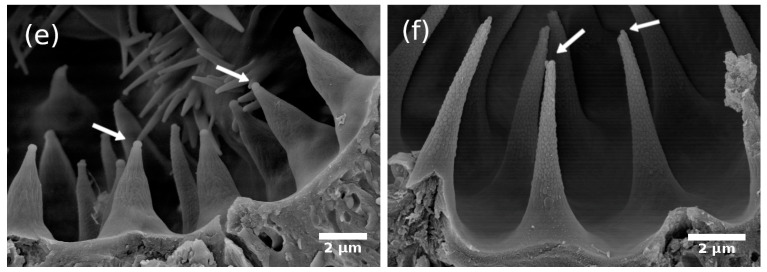
Scanning electron micrographs of the structures in the sensory pit located in the flagellum of the *A. ludens* antenna: (**a**) Internal part of the antenna showing the two chambers of the sensory pit; (**b**) Close-up of the chambers showing the structures (non-uniform wall and modified microtrichia pointed with white arrows) between the chambers. The distribution of the three subtypes of pit-sensilla is also discernible; (**c**) Internal chamber showing the distribution of pit-basiconic sensillum subtype I [pb-I] and II [pb-II]; White arrows point to non-uniform wall and modified microtrichia; (**d**) Close-up of pit-styloconic sensilla; (**e**) Close-up of pit-basiconic subtype I sensilla [pb-I] which have nutshell-like cuticle texture, nipple-like shape, and a tip protuberance (pointed by white arrows); (**f**) Close-up of pit-basiconic subtype II sensilla [pb-II], which have scaly-like cuticle texture with a rosette (pointed by white arrows) at the tip that appears to open and close.

Based on the shape, length, cuticle thickness, pore density in the cuticle (i.e., multiple, few, one, or none), pore shape, and if the sensillum is socket-based, we identified a total of 16 different sensilla subtypes (Figure 2, Figure 3, Figure 4, Figure 5, Figure 6, Figure 7, Figure 8, Figure 9, Figure 10, Figure 11 and Figure 12) (including the three sensilla in the sensory pit) and microtrichia (mi), mainly distributed on the flagellum (Figure 1b,c, Video S1; Table 2) of the antennae of *A. ludens* females and males.

On the scape, we only detected chaetica sensilla (ch) and microtrichia (Figure 1a). The pedicel has a line of prominent chaetica sensilla in the frontal margin and plenty of microtrichia (Figure 1a).

On the flagellum or funiculus, based on the shape and using TEM and SEM techniques, we identified four main types of sensilla—trichoid (tr), clavate (c), basiconic (b), and styloconic (s)—with different subtypes according to the presence and shape of pores, cuticle width and size (Figure 2, Figure 3, Figure 4, Figure 5, Figure 6, Figure 7, Figure 8, Figure 9, Figure 10, Figure 11 and Figure 12). The four types were already reported for *A. ludens* by Dickens et al. [5] using different terminology (Table 2). In the TEM study, we also found a different kind of campaniform-like sensilla (cm), which was not previously reported for *A. ludens*. However, since we were unable to conclusively identify it in the SEM study, we handled this finding with caution because it could be an incomplete capture of a sensillum in a bad position. In addition, we report, for the first time, two subtypes of sensilla that differ from all previously described sensilla, inside of the sensory pit of the *A. ludens* flagellum. Below, we provide descriptions of each subtype of sensilla.

### 3.1. Types, Subtypes, and Descriptions of Sensilla

#### 3.1.1. Basiconic

The basiconic sensillum is digitiform, with a wide base that gradually narrows towards the tip and is shorter than the trichoid sensillum (Figure 2 and Figure 3).

We identified six subtypes of basiconic sensilla. According to their tip shape (sharp or blunt), longitude, the thickness of the cuticular wall, and the presence of hourglass-shaped pores in the flagellum, we recognized four subtypes along the flagellum (Figure 2 and Figure 3) and two inside of the sensory pit of the flagellum (check Section 3.2). Basiconic type I (b-I) are apparently the longest. They have a thin wall with hourglass pores, a rounded tip, and are inserted in a socket (Figure 2a,b and Figure 3a); this sensillum was named “thin-walled multipore pitted sensilla” (MPS) by Dickens et al. [5] and “thin-walled MPS long subtype I” by Castrejón-Gómez and Rojas [20].

Basiconic subtype II (b-II) sensilla are shorter and with a bigger diameter in the tip than b-I, have a thin cuticular wall with hourglass pores, a rounded tip, and are also inserted in a socket (Figure 2a,c and Figure 3b); this sensillum was named “thin-walled MPS short subtype II” by Castrejón-Gómez and Rojas [20].

Basiconic subtype III (b-III) sensilla have a thick cuticular wall with few wedge-shaped pores (Figure 2a,d and Figure 3c). Finally, basiconic subtype IV (b-IV) sensilla are the smallest of our sub-classification, have a socket, a thick cuticular wall, and few wedge-shaped pores (Figure 2a,e and Figure 4e–f).

#### 3.1.2. Chaetica Sensilla

The chaetica sensilla are the longest in the antenna, with a cone shape. They are longitudinally ridged and have a pointed tip (Figure 5). The end of the ridged part of the hair is attached to a socket that is probably suspended in a joint membrane [2], which permits the sensillum’s free movement with an apparent mechanoreception function (Figure 5c,d). Interestingly, in the lower outside part of the socket, there is a group of 16–20 tiny pores (Figure 5c,d).

#### 3.1.3. Clavate Sensilla

According to the sensillum shape, the width of the cuticle wall, and the pore shape in the cuticle, we defined four subtypes of clavate sensilla (Figure 6 and Figure 7); one more than reported for other species of tephritids to date.

The clavate type I (c-I) sensilla have a short waist, which widens at the top, and a thick cuticular wall (Figure 7a), probably with wedge-shaped pores, similar to those shown in Figure 4e,f. The clavate-type II (c-II) sensilla, observed on the medium part of the female flagellum, have the smallest diameter in the middle part, are socket-based, and have a thin wall (Figure 6c,d and Figure 7b) with an hourglass-shape and multiple similar to those shown in Figure 4a,d. The clavate type III (c-III) sensilla have the biggest diameter and thickest walls, apparently with pores (Figure 7c). This sensillum, which is reported for the first time in a tephritid fly, has a typical club shape, and a shorter base than subtypes I, II and IV; it was only observed in the distal flagellum of females and males. Finally, we identified a clavate type IV (c-IV) sensillum through TEM with a similar shape to subtype I but exhibiting a thinner wall with multiple hourglass-shaped pores (Figure 7d).

#### 3.1.4. Styloconic

These sensilla are the smallest ones we identified. They are about 3 µm long and are characterized by grooves and ridges that make them look like a group of digitiform projections (Figure 8). They have a double wall (Figure 4c) that consists of a cuticular sheath that wraps three dendrites in its internal lumen. They have 11-digit type projections with pores between them (Figure 8a,b). We detected them in different areas of the flagellum of males and females.

#### 3.1.5. Trichoid

The multipore trichoid-type sensilla we identified had the longest, thinnest, and most conspicuous shape of all sensilla identified in this study (Figure 9 and Figure 10), similar to those previously reported for *B. tryoni* [13,16], *A. curvicauda* [11], and *A. fraterculus* [7]. In our case, however, we identified two subtypes of trichoid sensilla (Figure 9 and Figure 10).

In observations of longitudinal sections via TEM, we detected that within the trichoid sensilla, there were two variants: sharp (Figure 9 and Figure 10a), and blunt-tipped (Figure 9 and Figure 10b), which we named trichoid I (Tr-I) and trichoid II (Tr-II), respectively. Both types are thin-walled with an hourglass shape and multiple pores (Figure 10a,b). In Figure 4a, a close-up of the trichoid sensillum of the proximal flagellum, it is possible to perceive the hourglass-shaped pores and the tubule projections that run into the dendrite branches. Tr-I are the most abundant and longest in the flagellum and are longer than Tr-II (Figure 9a).

#### 3.1.6. Campaniform-like Sensillum and Glands

In the flagellum of an *A. ludens* male, we identified, with the help of TEM images, campaniform-like sensilla (Figure 11). In this case, three long cells were observed behind the cuticula, clustered very close to the sheath surrounding the sensilla’s dendrite, like a campaniform sensillum, which could possibly be a secretory cell associated with this sensillum (Figure 11a).

In the TEM images, we also observed campaniform-like sensilla in females, which have a flattened external cuticular area (there is no hair as such) and are apparently innervated by two sensitive cells. Moreover, this type of sensillum is found very close to a group of secretory cells directly in contact with the cuticula, where numerous channels can be observed (Figure 11b–d). Unfortunately, we were not able to identify these sensilla with SEM images.

### 3.2. Sensory Pit

The olfactory or sensory pit is located in the dorsobasal part of the antenna (Figure 12a, Video S2), and it is composed of two chambers (internal and external) where we found three subtypes of sensilla (Figure 12b). Chambers are physically semi-separated by a bridge of structures that look like modified microtrichia of different sizes and shapes, as well as modifications of the cuticular floor (Figure 12b,c).

**External chamber (Ec):** This chamber is the outermost in the pit and the smallest. It has at least eight styloconic sensilla (Figure 12b), which have, from the middle towards the tip, the characteristic finger-like form of the styloconic sensillae with different longitudinal fingers, and have a smooth cuticle from the middle towards the base (Figure 12d). We named them pit-styloconic sensilla (ps). They are longer (ca. 7µm long) than the styloconic sensilla found in the rest of the flagellum (ca. 3.76 µm long) and are inserted into sockets, either alone or in pairs. This type of sensillum is like the one reported as “grooved sensillum” by Honda et al. [26] in the “large olfactory pit” of the onion fly, *Hylemya antiqua* Meigen (Diptera: Anthomyiidae). Although these authors suggested an olfactory function, we detected no apparent pores in the cuticle, so their function remains uncertain.

**Internal chamber (Ic):** This chamber is bigger than the external one and has two subtypes of basiconic sensilla, which are different from all sensilla in the rest of the flagellum. We named them pit-basiconic sensilla type I (pb-I) (Figure 12e) and type II (pb-II) (Figure 12f).

**Figure 13:**
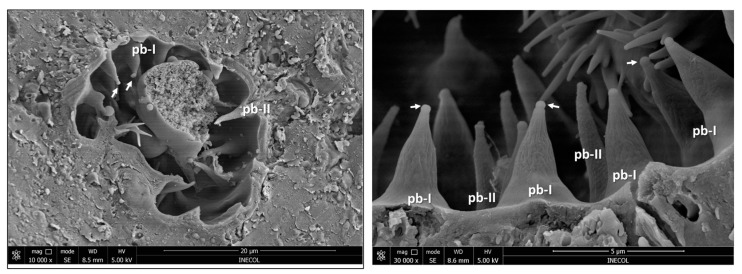
Scanning electron micrographs of the internal chamber of the sensory pit with pb-I and pb-II sensilla. Short arrows show the tip of pit-basiconic subtype I [pb-I] with a kind of porous plug, which could also be a secretion of viscous fluid containing a mucopolysaccharide that usually covers the tips of chemoreceptor dendrites and sometimes is exuded through the terminal pore [1].

There are about 20 pit-basiconic-type I sensilla (with a nipple-like shape) (Figure S1), mainly located on the proximal side to the base of the antenna (Figure 12b). They are approximately 3–4 µm long, have a nutshell-like cuticle texture in two-thirds of the sensillum cuticle from the tip to the base, are socketed, and end with a spherical protuberance or a kind of porous plug (Figure 12e and Figure 13).

The pit-basiconic type II (pb-II) sensilla are about 6 µm long. Approximately 13–15 pb-II are located on the side facing the apical end of the antenna (Figure 12b). They have a scaly-like texture with a rosette at the tip that appears to open and close (Figure 12f). This sensillum is similar in shape to the “striated pit sensillum” reported by Honda et al. [26] in the “large olfactory pit” of the onion fly. These authors reported, for the “striated pit sensillum”, the presence of two sensory neurons that extend their dendrites to the sensillum tip, but they did not observe any pores or opening in the tip that could have suggested a gustatory or olfactory function. In our case, we do not have an internal image of this sensillum showing the dendrites, but we have images suggesting that the rosette in the tip could be a type of mouth that opens and closes. Considering the similitude of the pit-basiconic type II with the “striated pit sensillum” reported by Honda et al. [26], we suggest that this sensillum has an olfactory function.

### 3.3. Microtrichia (mi)

**Figure 14:**
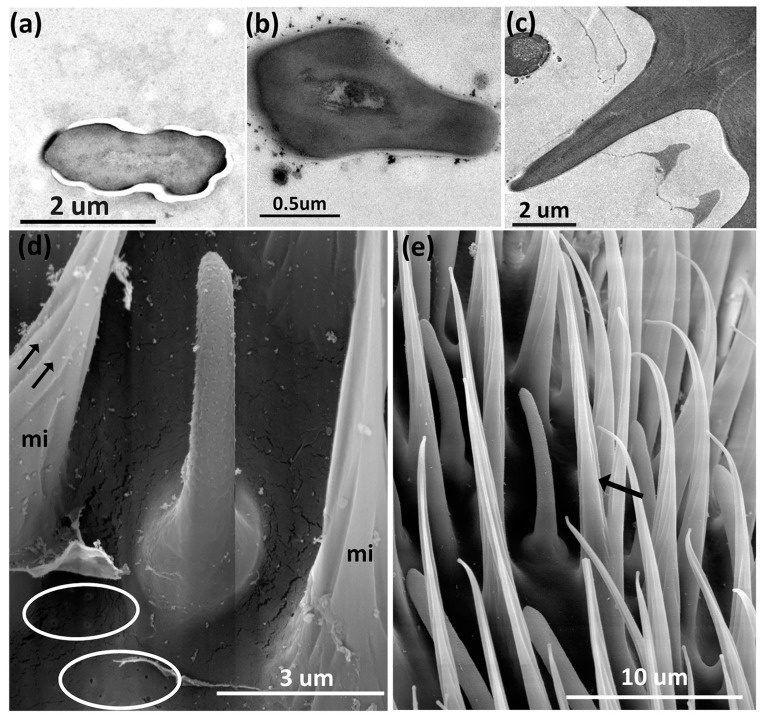
TEM and SEM of microtrichia: (**a**) TEM image of a transversal section of a microtrichium showing the lumen in the center; (**b**) TEM image of a transversal section of a microtrichium showing, in the center, a probable dendrite; (**c**) TEM of a microtrichium longitudinal section showing the lumen; (**d**) SEM of basiconic sensillum and microtrichia [mi] with some pores (inside white circles) in the antenna cuticle near their bases; black arrows point to the ridges of microtrichia; (**e**) Microtrichia (black arrow) surrounding sensilla.

These microstructures (“mi” in Figure 5b, Figure 6b, Figure 10a and Figure 14) distributed along the antenna are curved, grooved, long, and thin projections that narrow at their apical part, ending in a sharp point. These projections are non-innervated, as becomes apparent in Figure 10a and Figure 14a–c), and in line with what other authors have reported [5,16]. However, in a transversal cut made in the middle of a microtrichium, it is possible to observe what appears to be a dendrite (Figure 14b). We propose that microtrichia are likely more associated with a protective function of the sensilla in the antenna. We also suggest that the longitudinal ridges of microtrichia could help conduct some substances by runoff to the pores on the antenna cuticle (Figure 14d). Some of the microtrichia inside the sensory pit are modified and partially separate the two chambers (Figure 12b,c).

### 3.4. Other Structures

**Figure 15:**
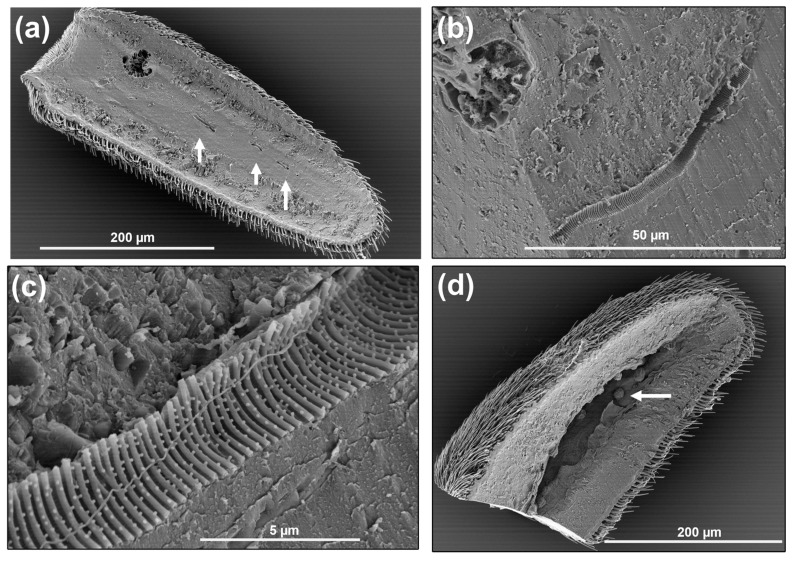
(**a**) The internal part of the antenna showing sections of the tracheal tube that passes through the antenna longitudinally (white arrows); (**b**) Cose-up of the trachea behind the sensory pit; (**c**) Close-up of the trachea formed by tubes (tubes were cut during sample preparation) with small pedicelled spherical protuberances and a duct crossing the antenna; (**d**) Internal part of the antenna showing several rough spherical structures.

In addition to sensilla, we report on other structures discovered during the preparation of the samples for SEM and TEM studies. For example, when the antenna was cut longitudinally, we found a tracheal tube crossing the medial–internal part (Figure 15a–c). Tracheae are part of the insect’s air supply system, and their abundance in specific body parts or tissues reflects the demand for oxygen in those parts [1]. Other structures that we found included several rough spherical structures distributed along the deeper medium part of the flagellum (Figure 15d). We also identified small pores on the antenna cuticle on the base of some sensilla and microtrichia (Figure 5c,d and Figure 14d).

## 4. Discussion

Considering the shape, size, wall thickness, and presence of pores in the cuticle and the location (flagellum and sensory pit), we were able to characterize and suggest the function of 16 subtypes of sensilla (13 in the flagellum and three in the sensory pit) of the *A. ludens* antennae. That is, we described 12 additional subtypes to those reported by Dickens et al. [5] who only characterized four types (Table 2). We report, for the first time in *A. ludens*: (a) two types of pores in the sensilla (hourglass and wedge shapes) that we also used to classify the sensilla; (b) the description of the sensory pit and their associated sensilla, classifying them according to their shape (to name them, we add the prefix “pit”); (c) the presence of two chambers (external and internal chambers); (d) pit-styloconic (ps) sensilla in the external chamber, and pit-basiconic subtype I (pb-I) and subtype II (pb-II) sensilla in the internal chamber (Figure 12); (e) a porous plug or secretion that apparently flows from the sensilla pit-basiconic subtype I (pb-I) (Figure 13); (f) a campaniform-like sensillum in the flagellum (although we could not confirm its presence with SEM images); (g) the presence of pores in the antenna cuticle (Figure 5c,d and Figure 14); and (h) the presence of a tracheal tube crossing the internal part of the antenna longitudinally (Figure 15).

The difference in the number of sensilla subtypes with respect to the ones reported by Dickens et al. [5] is partly because these authors based their classification on the terminology proposed by Altner [3], and thus only considered the presence/absence of pores and the thickness of the cuticle, classifying all types of sensilla as no-pore sensilla (NPS) and multiple pitted sensilla (MPS), and in the case of subtypes, thick (Thick-Walled MPS), thin (Thin-Walled MPS) and Multiporous Grooved Sensilla (MPGS) (Table 2). However, the complex repertoire of behaviors that *A. ludens* and other fruit flies exhibit during host and food location, as well as courtship and mating, suggests the existence of a more sophisticated group of sensilla than just four types.

The terminology used to describe and classify sensilla has been changing according to the development of microscopy techniques that now allow for us to detect/describe internal details that are helpful in better characterizing a sensillum. In our case, we partially used the old system of Schenk [27], based on the shape and mode of insertion in the antenna-cuticular wall (e.g., the presence of a socket), which is still practical when distinguishing one type from another with a light microscope. We also used a system used by other authors e.g., [3,5,28,29,30,31] based on the presence or absence of cuticular pores, a single (thin) or double (thick) cuticular-wall, as well as the study of other internal structures discovered with SEM and TEM techniques. These two approaches helped us considerably refine the classification used in the only other study on the antenna of *A. ludens* [5].

In the case of true fruit flies (Diptera: Tephritidae), most investigations have been restricted to the study/description of morphological structures in the sensilla of the antenna, and very few have tested the functionality of these structures. Considering that adult fruit flies follow odors as cues to find food, mates, and hosts, the great advantage and contribution of the work by Dickens and collaborators [5] was the inclusion of some electrophysiological tests with *C. capitata* that confirmed that sensilla with pores were related to chemoreception and those without pores were related to mechanoreception.

We found abundant microtrichia in the pedicel and flagellum of the *A. ludens* antenna, which coincides with the findings of Dickens et al. [5], and other authors such as Giannakakis and Fletcher [16], Bisotto de Oliveira et al. [7], Hu et al. [17], and Perre et al. [12], among others, who, working with other species of fruit flies, all reported that microtrichia constitute the major cuticular structures in the antenna compared with sensilla. However, most authors describe microtrichia as non-innervated setae, curved and longitudinally ridged, omitting any mention of their function, except for Hu et al. [17], who named them “microtrichial sensilla” inferring a mechanoreception function for six species of *Bactrocera*. However, in the latter study, it is not possible to observe the elastic membrane (the joining or socket membrane observed in the chaetica sensilla in Figure 5c,d of our study) that, according to Keil and Steinbrech [2], permits the sensillum movement and, with it, the stimulation of the outer dendritic segment that in mechanosensitive sensilla is only located in the internal base of the sensillum. In our case, we show innervated microtrichia without the elastic membrane, socket, and pores (Figure 10a and Figure 14a,c), which indicates that they do not have mechanical or chemoreceptive functions. However, we found probable evidence that microtrichia may have what appears to be a dendrite in the center, although we could not see the other usually related ultrastructures (Figure 14b). Since we could not see a well-developed dendrite in all samples, we suggest that the microtrichia could be vestiges of true sensilla that gradually lost their main function in the evolutionary specialization process of this group of flies and that, since they are very abundant and surround the true sensilla, they could possibly work as physical protectors of sensilla. We surmise that they could also possibly capture chemicals to avoid chemoreceptor sensilla saturation and conduct the captured chemicals through their longitudinal ridges to the base of the antenna cuticle, where some pores are present (Figure 5c–d and Figure 14). Also, since we did not observe pores in the microtrichia, we suppose that they do not have a chemoreceptive function.

The trichoid sensilla we found in *A. ludens* are similar to those reported in other fruit flies such as *B. tryoni* [13,16], *A*. *curvicauda* [11], *A. serpentina* [20], *A. fraterculus* [7,16], and the other eight species of *Anastrepha* [12]. Notably, the trichoid sensilla we observed were not reported by Dickens et al. [5], who mention “longitudinally ridged trichoid mechanosensory sensilla along the distal margins of both the scape and the pedicel” that they classified as “No-Pore Sensilla” but that we classified as Chaetica sensilla. The same authors refer to the arista as “an elongated trichoid arista”. In our study, Tr-I and Tr-II were found to be thin-walled with multiple hourglass-like pores, a detail not reported before; Tr-I are sharp, and Tr-II blunt-tipped (Figure 9 and Figure 10). Pore presence and the shape of Tr-I coincide with the trichoid type I reported for *B. tryoni* by Giannakakis and Fletcher [16]; the difference is that, in our case, we report that trichoid subtype I (Tr-I) have a thin wall and sharp tip (Figure 9a and Figure 10a), while those authors report a thick wall for trichoid type I. Trichoid subtype II is also similar to the trichoid type II reported by Giannakakis and Fletcher [16]. Trichoid sensilla are considered chemoreceptors and are associated with the behavior of orientation and intraspecific communication [2], specifically pheromone recognition [32,33]. In fruit flies, Levinson et al. [8] and Dickens et al. [5] reported that trichoid sensilla (= thick-walled-[MPS] of *C. capitata*) respond to sex pheromone and trimedlure extracts (an attractant based on sexual pheromones), respectively.

In the case of basiconic and clavate sensilla, there is great diversity in the *A. ludens* flagellum we studied; therefore, it is sometimes complicated to discern one from the other through SEM. These types of sensilla have been also reported in several other tephritid fruit flies such as *A. fraterculus* [7], *A*. *curvicauda* [11], *C*. *capitata* [8], *A*. *serpentina* [20], *B. tryoni* [13,16] and, very recently, in eight species of *Anastrepha* present in Brazil [12]. In the case of *B. dorsalis*, Liu et al. [34] did not find clavate sensilla in the antenna of this species. Despite the above, no specific studies on the function of these sensilla have been performed, although most of the previously cited authors suggest that basiconic and clavate are chemosensilla, mainly based on the presence of pores. Keil and Steinbrecht [2] mention that basiconic sensilla in *Bombyx mori* L. have a thin cuticular wall, higher pore density, a higher numbers of pore tubules per pore, and a greater number of dendrites than trichoid sensilla. Although these authors did not identify a functional role in the studied structures, based on the fact that *B. mori* basiconic sensilla respond to fatty acids and alcohols, and considering that other insects have basiconic sensilla with similar features, they suggest a possible function in food finding and selection.

In our observations, the hourglass pores were more related to thin cuticular-wall sensilla, and wedge or funnel-like pores were more related to thick cuticular walls, with both pore types presenting several tubules, which coincides with that reported by Keil and Steinbrecht [2]. These authors report that the pheromone-sensitive trichoid sensilla of *B. mori* have thick walls and funnel or wedge-shaped pores, which have a narrow channel with tubules running from the channel to a broader fluid-filled canal to contact the dendrites. Considering this, we surmise that the pores in the trichoid sensilla, the longest ones (Figure 1b,c and Figure 9a) in the flagellum, could help transport pheromones to the dendrites.

With the help of TEM, we observed probable secretory epithelial cells contiguous to the cuticle (class 1 glands) associated with campaniform-like sensilla, which had not been described in the antenna of *A. ludens* and other fruit flies, except in *B. zonata*. In this case, Awad et al. [18] reported the presence of campaniform sensilla on the pedicel of males and suggested that they are mechanoreceptors. The glands on antennae in both males and females, first discovered via TEM, have been widely described in egg parasitoids associating Type 1 glands with campaniform sensilla [35]. Usually, campaniform sensilla are located in structures where a mechanical deformation occurs on the cuticle [2,36]. In our case, we could not identify these glands with the help of SEM as campaniform sensilla in the flagellum are surrounded by many microtrichia and other sensilla, which made it difficult to find them.

We report a styloconic sensilla (Figure 8) distributed along the pedicel of females and males. This sensillum type is similar to those reported with the same name by Giannakakis and Fletcher [16], Arzuffi et al. [11], Bissotto de Oliveira et al. [7], and referred to as multiporous grooved sensilla (MPGS) by Dickens et al. [5] and Castrejón and Rojas [20], grooved sensilla by Mayo et al. [9] and Bigiani et al. [10], or coeloconic sensilla by Keil and Steinbrech [2] and Awad et al. [18], among other authors. In other insect species, in the case of this sensillum type, chemoreception [13,16], higroreception and thermoreception functions have been reported [3].

We found that the sensory pit in the *A. ludens* antenna has two chambers, the external (most outer) and the internal, with distinct types of sensilla in each one (Figure 12). The external chamber has a group of pit-styloconic sensilla, which coincides with the only sensory pit chamber reported in *B. zonata* [18], where only styloconic sensilla are found. Styloconic sensilla are also found in the “large pit” of the onion fly (*H. antiqua*), with the difference that, in that chamber, there are two subtypes of sensilla [26]. In both cases, the authors propose an olfactory function for these sensilla.

The internal chamber of *A. ludens* is like “Chamber III” of *Drosophila melanogaster* Meigen (its sensory tip has three chambers), which has two types of sensilla [37]. However, the sensilla are quite different. In *A. ludens*, the pit basiconic type I (pb-I) sensillum is similar to the “no-pore coeloconica sensilla (np-CS)” in “Chamber II” of the *D. melanogaster* sensory pit because both have a kind of protuberance at the tip (Figure 12e and Figure 13) and a conical shape; however, they differ in the cuticular wall, which is smooth in the np-SC in *D. melanogaster* and nutshell-like in the pb-I (Figure 12e and Figure 13) of *A. ludens*. Shanbhag et al. [37] indicate that the protuberance of np-SC is the molting pore of the sensillum and that the lumen of the peg is filled with the dendritic outer segment of two sensory neurons and with electron-dense material. In our case, the protuberance in the pb-I (Figure 12e and Figure 13) could be a porous plug separating the dendrite ends from the environment, or it could also be part of a secretion of viscous fluid containing a mucopolysaccharide, which covers the tips of the contact chemoreceptor dendrites and is sometimes exuded through the terminal pore of the sensillum [1]. Honda et al. [26] reported a similar sensillum with a protuberance at the tip and elongated pores in the “small olfactory pit” of the onion fly, but this fly species has one large sensory pit and several (8–10) small olfactory pits.

The sensillum pit basiconic type II (pb-II) we describe is similar to the “striated pit sensillum” of the *H*. *antiqua* “large chamber” [26] and to the “grooved sensilla 1 and 2 (GS1 and GS2)” of *D. melanogaster* in the “Chamber III” [37]. These sensilla have an open slit channel system that permits access to the external environment, as is probably the case in the sensilla pb-II of *A. ludens* (Figure 12f). In *D. melanogaster,* Shanbhag et al. [37], considering the internal structure of this sensillum type, suggested a combination of olfactory and thermoreceptive functions.

Finally, we report some structures observed in the internal part of the antenna (Figure 14 and Figure 15) that will need to be studied in more detail to discover their function.

## 5. Conclusions

In conclusion, our data suggest that the antenna of *A. ludens* contains a complex group of chaetica, trichoid, clavate, basiconic, styloconic, and campaniform-like sensilla that likely participate in the perception of volatiles originating from congeners, host plants and food sources, as well as mechanoreception, thermoreception, and hygroreception. These functions need to be confirmed via electrophysiological, neurological, and behavioral studies, but an important step towards updating the knowledge on the antenna of *A. ludens*, a key pest of fruit in the Americas, has been achieved here. We also need to confirm if the various subtypes of sensilla identified here have different functions or simply represent natural variabilty in shape.

## Figures and Tables

**Table 1:** Mean (± SE) length and width of different antennal segments of *A. ludens* females and males (n = 50).

Segment	Length (µm)		T-Value	Width (µm)		T-Value
	Female	Male	(*p*-Value)	Female	Male	(*p*-Value)
Scape	97.3 ± 1.8	96.6 ± 1.5	0.31 (0.76)	180.9 ± 3.2	178.4 ± 2.0	0.66 (0.51)
Pedicel	169.5 ± 2.8	166.0 ± 2.4	0.94 (0.35)	193.2 ± 1.9	195.6 ± 1.5	−0.98 (0.33)
Flagellum	432.3 ± 3.3	430.5 ± 2.7	0.44 (0.66)	225.2 ± 4.5	214.8 ± 2.2	2.08 (**0.04**)
Arista	995.3 ± 4.7	998.8 ± 4.0	−0.55 (0.58)	38.36 ± 1.3	39.0 ± 1.6	−0.33 (0.74)

(*p*-Value) in bold numbers are significantly different (*p* < 0.05).

**Table 2:** Equivalencies between the names of the sensilla in the *A. ludens* flagellum used by us and the ones by Dickens et al. [5], including their putative/potential function.

No.	Sensillum Name Used Here	Sensillum Name *sensu* Dickens et al. [5]	Putative Function and (Location)
1	Basiconic I (rounded tip, the longest with socket, thin-walled multipore (MPS) with hourglass-like porous)	Thin-walled-MPS	Chemoreception (flagellum)
2	Basiconic II (rounded tip, shorter and with a bigger diameter than b-I with socket, thin-walled MPS with hourglass-like porous)	Not reported	Chemoreception (flagellum)
3	Basiconic III (thick-walled with few wedge-like pores)	Not reported	Chemoreception (flagellum)
4	Basiconic IV (smallest, in socket, thick-walled, with wedge-like porous	Not reported	Chemoreception (flagellum)
5	Pit-basiconic I (nutshell-like cuticle texture with tip protuberance)	Not reported	Contact chemoreception (Internal chamber of the sensory pit)
6	Pit-basiconic II (scaly-like cuticle texture with a tip rosette)	Not reported	Chemoreception/Thermoreception (Internal chamber of the sensory pit)
7	Campaniform-like	Not reported	(flagellum)
8	Chaetica	No-pore (NPS)	Mechanoreception (scape and pedicel)
9	Clavate I (thick-walled, wedge-like pore shape)	Not reported	Chemoreception (flagellum)
10	Clavate II (thin-walled, MP with hourglass-like pore shape and with socket)	Not reported	Chemoreception (flagellum)
11	Clavate III (the shortest, thick-walled, and without pores)	Not reported	Mechanoreception (flagellum)
12	Clavate IV (similar to C-I shape but thin-walled and hourglass-like pore shape)	Not reported	Chemoreception (flagellum)
13	Styloconic	Multiporous grooved sensilla MPGS	Chemoreception (flagellum)
14	Pit-styloconic	Not reported	(External chamber of the sensory pit)
15	Trichoid I (thin-walled, hourglass-like pores and sharp-tipped, the longest of flagellum)	Thick-walled-MPS	Chemoreception (flagellum)
16	Trichoid II (thin-walled, hourglass-like pores and blunt-tipped)	Not reported	Chemoreception (flagellum)

## Data Availability

Data are contained within the article or Supplementary Material.

## Supplementary Materials

The following supporting information can be downloaded at: https://www.mdpi.com/article/10.3390/insects14070652/s1, Figure S1: Scanning electron micrograph of the internal chamber of the sensory pit showing the distribution of at least 20 pit-basiconic type I (pb-I) sensilla, identified by white asterisks; Video S1: Three-dimensional view using confocal microscopy of the sensilla and microtrichia of *Anastrepha ludens*; several sizes of sensilla and microtrichia with forked tips are shown (depth coding mode); Video S2: Three-dimensional rotational view of the sensory pit of *Anastrepha ludens*; green color represents autofluorescence of the cuticle (447–543 nm) with 405 nm laser excitation, and red color represents chitin stained with Congo Red.
