# Supplementary material for: New Insights on Antennal Sensilla of Anastrepha ludens (Diptera: Tephritidae) Using Advanced Microscopy Techniques
Source: Insects. 2023 Jul 20;14(7):652. doi: 10.3390/insects14070652 (PMC10380199; doi:10.3390/insects14070652)
Supplement: Supplementary file 1 [file insects-14-00652-s001.zip › Figure S1.pdf]

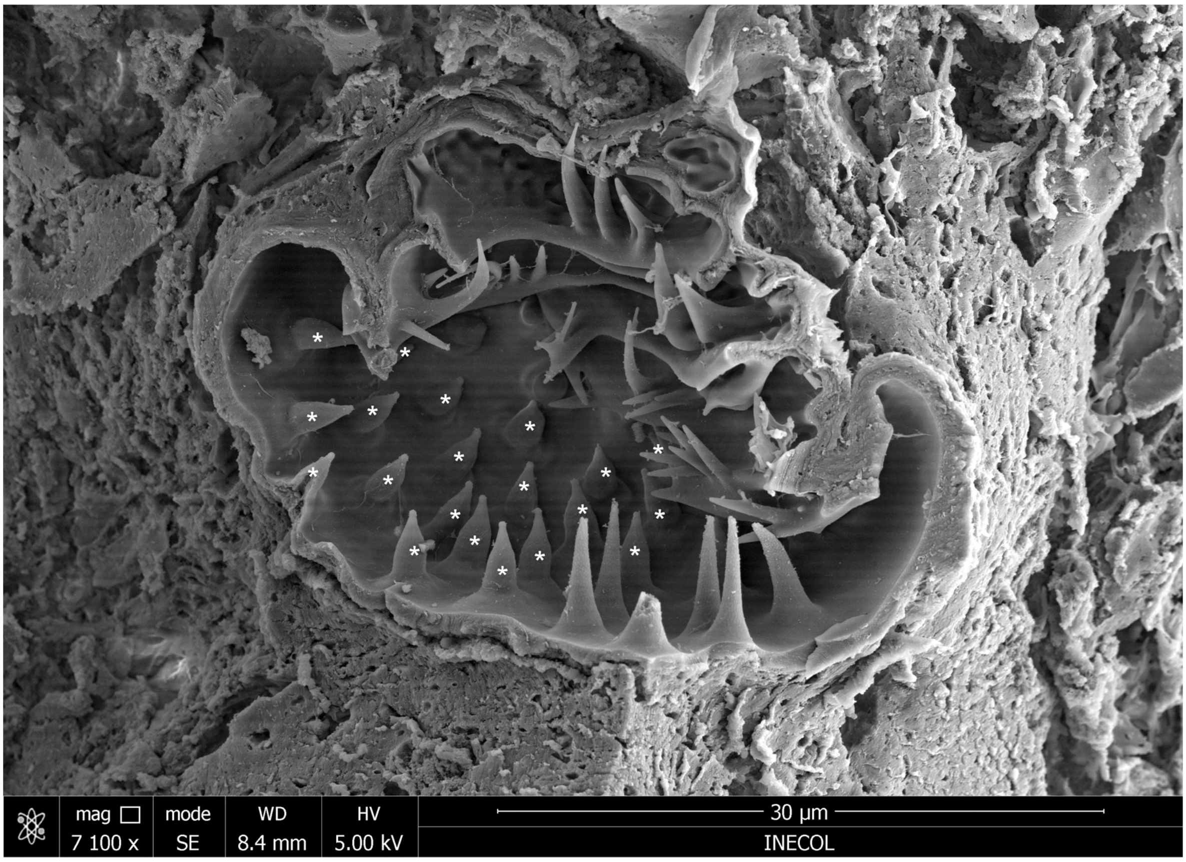

Figure S1. Scanning electron micrograph of the internal chamber of the sensory pit showing the distribution of at least 20 pit-basiconic type I (pb-I) sensilla, identified by white asterisks.
